# First-principles investigation of mechanical properties and fracture toughness in doped FeNbSb half-Heusler alloys

**DOI:** 10.1016/j.isci.2025.114189

**Published:** 2025-11-22

**Authors:** Shao-Bo Chen, Jia-Hao Wang, Wen-Jing Liu, Wan-Jun Yan, Ting-Hong Gao

**Affiliations:** 1 College of Electronic and Information Engineering, Anshun University, Anshun 561000, P.R. China; 2 Institute of Advanced Optoelectronic Materials and Technology, College of Big Data and Information Engineering, Guizhou University, Guiyang 550025, P.R. China

**Keywords:** Physics, Mechanical phenomenon, Applied sciences

## Abstract

The ductile-to-brittle behavior of thermoelectric half-Heusler alloys is critical for their mechanical reliability. This study investigates the effect of Co/Ni doping on the fracture toughness of FeNbSb using first-principles calculations. All doped derivatives considered in this study, except Fe_4_NiNb_3_Sb_4_, exhibit dynamic and thermodynamic stability. While doping generally improves ductility, the fracture toughness, derived from Griffith’s theory, reveals a strong site dependence. Co substitution at Fe or Nb sites raises the toughness to 2.043 MPa m^1/2^, exceeding the intrinsic value of 1.964 MPa m^1/2^. Conversely, Ni doping and Co at Sb sites reduce the crack propagation resistance. This variation is governed by an atomic-size-effect strategy, where dopants with larger radii enhance toughness, and is correlated with electronic structure evolution near the Fermi level. The findings provide a rationale for designing damage-tolerant half-Heusler materials for thermoelectric and high-temperature applications.

## Highlights

• First-principles study of FeNbSb and Co/Ni-doped derivatives reveals mechanical properties
• Co-doping at Fe/Nb enhances fracture toughness to 2.043 MPa m^1/2^
• Atomic-size effect governs toughness, providing rationale for damage-tolerant alloy design

## Graphical abstract

**Figure:**
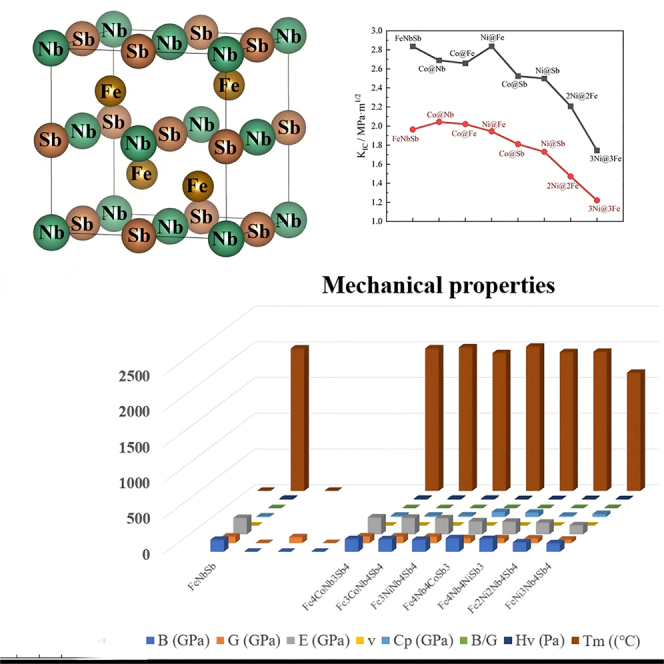

## Introduction

The pursuit of advanced materials for critical applications in aerospace, energy, and manufacturing increasingly demands a dual focus on functional performance and structural reliability. Metal-intermetallic composites and complex alloys, such as half-Heusler (HH) phases, are at the forefront of this endeavor, offering a unique combination of functional properties and high-temperature strength.^1^^,^^2^^,^^3^^,^^4^ For practical applications, especially in high-temperature and structural environments, superior thermoelectric conversion efficiency must be coupled with outstanding mechanical reliability. The mechanical integrity of these materials, particularly their resistance to fracture and crack propagation, is critical to ensuring device longevity and operational stability. However, a pervasive challenge for many intermetallics is their inherent tendency toward brittle fracture, which severely limits their service life and damage tolerance under mechanical stress.^5^^,^^6^ Overcoming this brittleness without compromising other desirable properties remains a central challenge in physical metallurgy and materials design. Traditional strategies to toughen intermetallic compounds often involve elemental doping or alloying. Yet, predicting the outcome of such interventions has largely relied on empirical rules or semi-empirical criteria derived from bulk elastic properties, such as Pugh’s ratio (*B*/*G*) and Poisson’s ratio (*v*).^7^ While these metrics provide valuable initial screening, they offer an incomplete picture. They fail to capture the atomistic mechanisms governing crack propagation—the ultimate determinant of fracture toughness (*K*_IC_), which is the critical property defining a material’s resistance to catastrophic failure.^8^^,^^9^^,^^10^ A fundamental, mechanistic understanding of how specific doping elements and their lattice site selection influence intrinsic fracture pathways is, therefore, essential to move from empirical tuning to rational design of damage-tolerant materials.

In the HH alloy system, FeNbSb^11^^,^^12^^,^^13^ has attracted much attention. FeNbSb is a typical p-type semiconductor, and its unique crystal structure provides room for performance optimization. Through doping,^14^^,^^15^^,^^16^ the carrier concentration can be precisely regulated, the electron transport efficiency can be significantly improved, the thermal conductivity can be reduced, and the thermoelectric properties can be effectively optimized, showing great potential in high-temperature thermoelectric power generation fields such as industrial waste heat recovery. While numerous studies have focused on optimizing the electronic and thermal properties of FeNbSb through elemental doping,^17^^,^^18^ a systematic investigation into the resulting mechanical properties, especially *K*_IC_ and its underlying toughening mechanisms, remains considerably less explored.

This study employs the FeNbSb HH system as a model platform to decode the atomistic principles of *K*_IC_ manipulation by isovalent transition metal doping (Co, Ni). We posit that the mechanical response of a complex alloy to doping is not arbitrary but is governed by a fundamental atomic-size-effect strategy. To test this hypothesis, we transition from traditional elasticity-based assessments to a direct, energy-based calculation of the critical stress intensity factor. Using first-principles calculations, the dynamic and thermodynamic stability of various doping configurations were systematically evaluated. We then decoupled the effects of ductility (via elastic moduli) from intrinsic *K*_IC_. Our results reveal a striking site-dependent duality: certain dopant-site combinations (e.g., Co doping at Fe/Nb sites) act as toughening agents, while others (e.g., doping at Sb sites) lead to embrittlement. Crucially, we demonstrate that this behavior is primarily orchestrated by the atomic radius of the dopant relative to the host atoms, a simple yet powerful predictive parameter. Our results provide important corroboration that the atomic-size-effect strategy is a viable and effective approach for enhancing *K*_IC_ in intermetallic alloys. The mechanistic insights gained here, particularly the crucial role of dopant atomic radius relative to host atoms, offer a valuable reference and guidance for future studies aimed at toughening other brittle material systems.

## Results

### Crystal structure and stability

**Figure 1:**
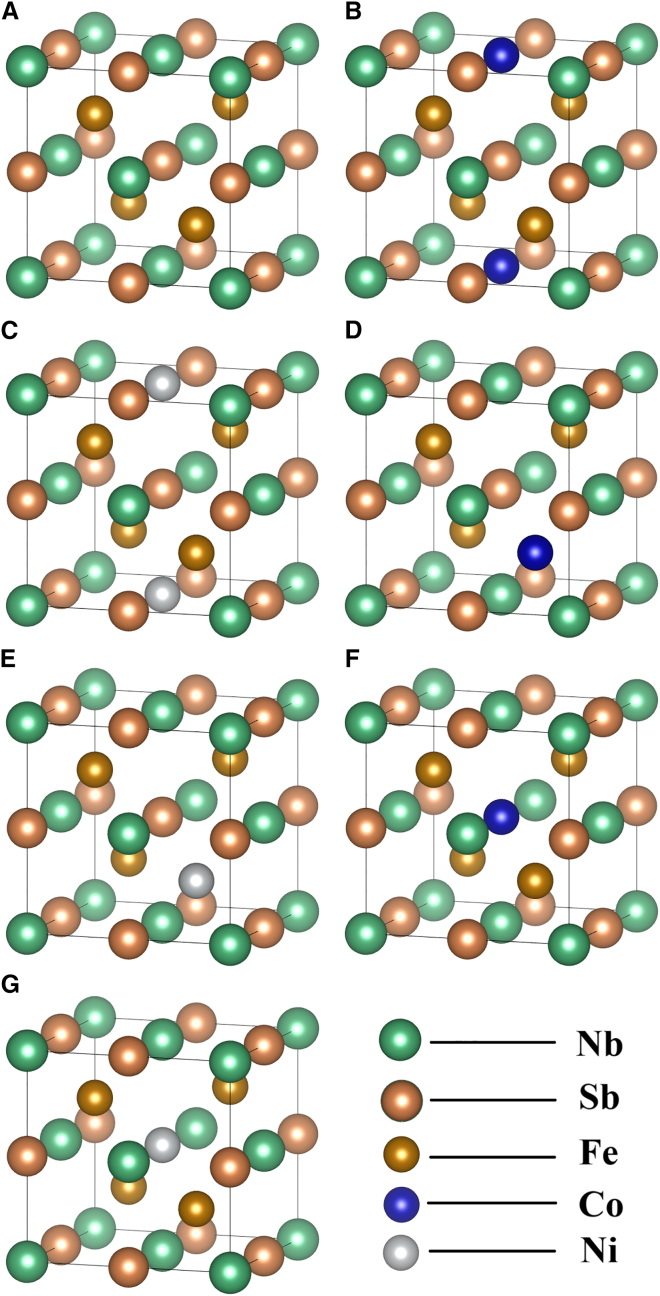
Schematic diagrams of the crystal structures of half-Heusler FeNbSb and its derivatives doped with Co and Ni elements (A) Crystal structure of intrinsic FeNbSb. (B) Crystal structure of intrinsic Fe_4_CoNb_3_Sb_4_. (C) Crystal structure of intrinsic Fe_4_NiNb_3_Sb_4_. (D) Crystal structure of intrinsic Fe_3_CoNb_4_Sb_4_. (E) Crystal structure of intrinsic Fe_3_NiNb_4_Sb_4_. (F) Crystal structure of intrinsic Fe_4_Nb_4_CoSb_3_. (G) Crystal structure of intrinsic Fe_4_Nb_4_NiSb_3_.

As illustrated in Figure 1A, the crystal structure of intrinsic FeNbSb belongs to the F-43m space group (No. 216), with a total of 12 atoms per unit cell. FeNbSb was modeled with the following Wyckoff positions: Nb at 4a (0, 0, 0), Sb at 4c (1/2, 1/2, 1/2), and Fe at 4b (1/4, 1/4, 1/4).^19^ The HH alloy XYZ structure^20^ adopts a cubic crystal system composed of three distinct elements (X, Y, Z). In this configuration, X atoms occupy the face-centered cubic (FCC) positions, Y atoms reside in tetrahedral interstitial sites, and Z atoms fill the octahedral interstitial sites, forming a uniquely ordered arrangement as illustrated in Figure 1A. Figures 1B–1G illustrate the derivative of the FeNbSb alloyed with Co or Ni atoms, in which the Co or Ni atoms replace the Fe atom, the Nb atom, or the Sb atom in intrinsic FeNbSb. The six faces of the unit cell contain two types of atoms: Nb and Sb atoms or Ni and Sb atoms, arranged in an alternating pattern, forming a complex lattice. Each subset of atoms independently constitutes a FCC Bravais lattice, resembling NaCl-type structure. This distinctive atomic architecture endows the alloy with exceptional physicochemical properties, demonstrating significant application potential in thermoelectric and magnetic fields. The lattice parameters obtained from geometric optimization are listed in Table 1. Notably, the lattice structure of intrinsic FeNbSb exhibits close agreement with reference data, validating the rationality of our computational methodology.

**Table 1:** Summarized lattice constants, formation enthalpy *E*_Formation_, site occupation energy *E*_site_, and dynamical stability

Crystal structure types	*a* = *b* (Å)	*E*_Formation_ (eV/atom)	*E*_site_ (eV/atom)	Dynamical stability
FeNbSb	5.933 5.9505^21^ 5.959 ^22^ 5.928 ^23^ 5.9136^24^	−0.7019	–	Yes
Fe_4_CoNb_3_Sb_4_	5.89155	−0.5378	−0.048	Yes
Fe_4_NiNb_3_Sb_4_	5.90258	−0.4898		No
Fe_3_CoNb_4_Sb_4_	5.94263	−0.6635	−0.1025	Yes
Fe_3_NiNb_4_Sb_4_	5.95683	−0.5610		Yes
Fe_4_Nb_4_NiSb_3_	5.87813	−0.3998	−0.0261	Yes
Fe_4_Nb_4_CoSb_3_	5.88548	−0.4259		Yes

**Figure 2:**
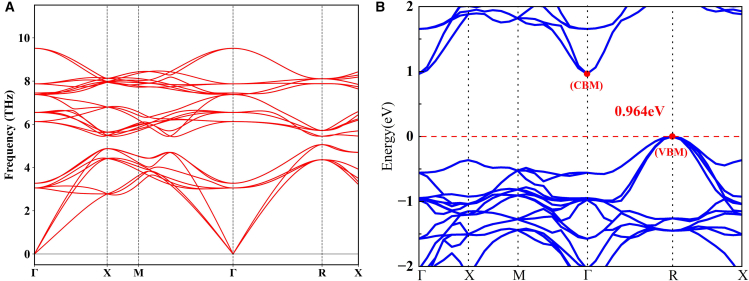
The calculated phonon spectrum and band structures of intrinsic FeNbSb (A) Phonon spectrum of the FeNbSb. (B) Band structure of the FeNbSb. The VBM and CBM are indicated by red dots.

**Figure 3:**
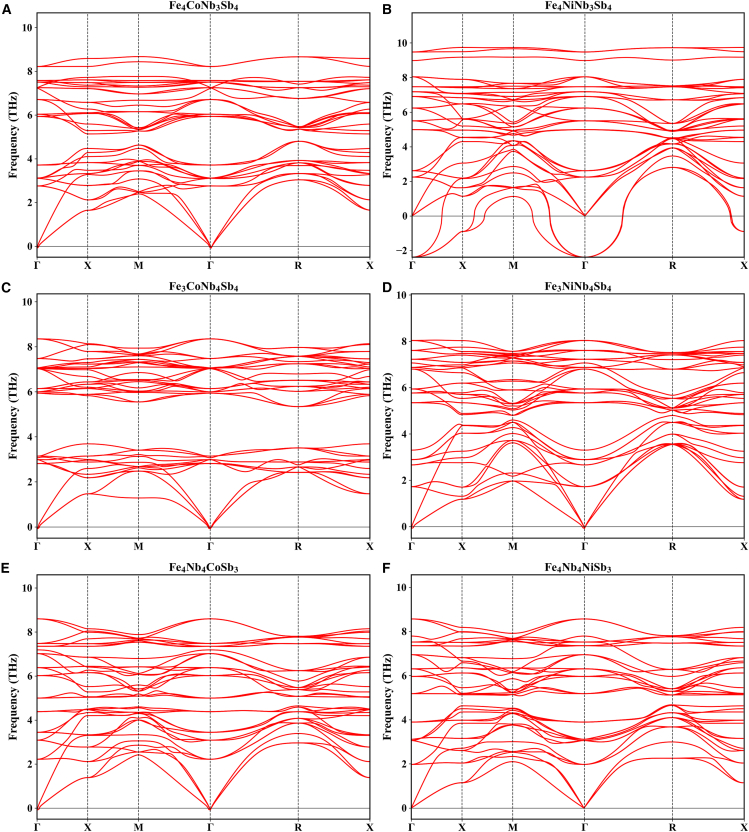
The summarized phonon spectrum of the derivative FeNbSb alloyed with Co and Ni atoms (A) The phonon spectrum of Fe_4_CoNb_3_Sb_4_. (B) The phonon spectrum of Fe_4_NiNb_3_Sb_4_. (C) The phonon spectrum of Fe_3_CoNb_4_Sb_4_. (D) The phonon spectrum of Fe_3_NiNb_4_Sb_4_. (E) The phonon spectrum of Fe_4_Nb_4_CoSb_3_. (F) The phonon spectrum of Fe_4_Nb_4_NiSb_3_.

Prior to investigating material properties, material stability must be evaluated. The high-symmetry path employed in this study follows the Γ→X→M→Γ→R→X trajectory. The phonon spectrum of intrinsic FeNbSb is plotted in Figure 2A along high-symmetry paths in the first Brillouin zone of reciprocal space. The absence of imaginary frequencies in the phonon spectrum indicates that the intrinsic FeNbSb compound is dynamically stable and thus readily synthetically accessible in laboratory settings. The dynamical stability of six alloyed FeNbSb derivatives—with Co/Ni substitutions at individual Fe, Nb, and Sb sites—was assessed via phonon spectrum calculations (Figure 3). Except Fe_4_NiNb_3_Sb_4_ (where Ni substitutes one Nb atom), which lacks dynamical stability, the other five FeNbSb derivatives—Fe_4_CoNb_3_Sb_4_, Fe_3_CoNb_4_Sb_4_, Fe_3_NiNb_3_Sb_4_, Fe_4_Nb_4_CoSb_3_, and Fe_4_Nb_4_NiSb_3_—exhibit dynamical stability. Consequently, further investigations will target the five dynamically stable derivatives identified in Figure 3, excluding the metastable Fe_4_NiNb_3_Sb_4_ phase (see Table 1).

Furthermore, to examine the thermodynamic stability of doped materials, the formation enthalpy can be investigated. The formation enthalpy refers to the energy change when a compound is formed from its stable elemental constituents. A lower formation enthalpy indicates greater stability of the compound, with a more resistant structure and properties. This is because a low formation enthalpy corresponds to greater energy release during formation, resulting in a more stable low-energy state that is less prone to transformation into other substances. The formation enthalpy can be calculated using the following equation^25^^,^^26^:

(Equation 1) $$E_{\text{Formation}}=E_{tot}-\sum_{i}nE_{i}\text{,}$$

where *E*_tot_ represents the total energy of the system, *E*_i_ denotes the energy of the individual and isolated atom of the *i*-th element, and *n* indicates the number of atoms of the *i*-th element in the unit cell. The formation enthalpy is summarized in Table 1. This finding demonstrates that these compounds possess excellent thermodynamic stability. In addition, calculated formation enthalpies reveal that Co/Ni substitutions at Fe sites yield the most stable configurations after intrinsic FeNbSb, exhibiting minimal enthalpy values. This stability originates from the proximity in atomic radius and atomic number between Co/Ni dopants and host Fe atoms.

The site preference of dopant atoms is governed by their site occupation energy. The site preference of Co and Ni atoms occupying Fe, Nb, or Sb positions in FeNbSb directly dictates the stability of the doped matrix phase. Therefore, calculating the site occupation energy becomes critically important. The site occupation energy is given as,^27^

(Equation 2) $$E_{site}=H_{\text{Formation}}^{A}-H_{\text{Formation}}^{B}\text{,}$$

where $H_{\text{Formation}}^{A}$ and $H_{\text{Formation}}^{B}$ denote the formation enthalpies of Co and Ni occupying specific Fe (Nb, Sb) sites, respectively. If *E*_*site*_ < 0 Co atoms exhibit a stronger preference for occupying Fe (Nb, Sb) sites than Ni, conversely, Ni dominates when *E*_*site*_ > 0. Our calculations reveal that Co atoms preferentially occupy Fe (Nb, Sb) sites over Ni (See Table 1). Furthermore, comparative analysis of site preference energies demonstrates that both Co and Ni favor Fe sites most strongly, followed by Nb sites, with Sb sites being least preferred. This behavior primarily stems from the proximity in atomic radius and atomic number between Co/Ni and host Fe atoms.

**Figure 4:**
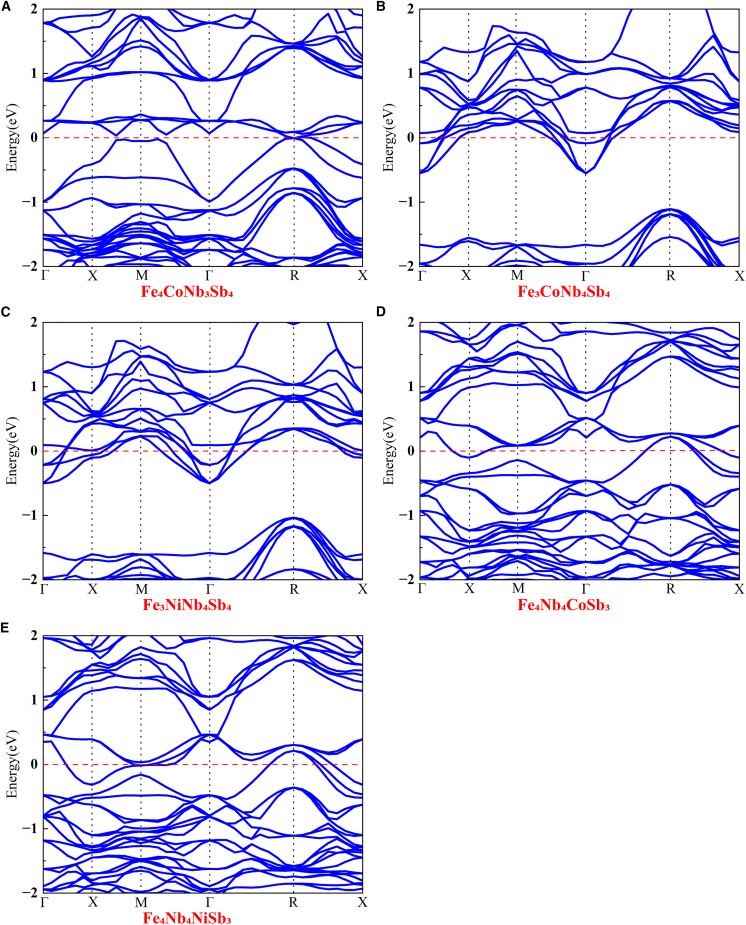
The summarized band structures of the derivative FeNbSb alloyed with Co and Ni atoms (A) The band structures of Fe_4_CoNb_3_Sb_4_. (B) The band structures of Fe_3_CoNb_4_Sb_4_. (C) The band structures of Fe_3_NiNb_4_Sb_4_. (D) The band structures of Fe_4_Nb_4_CoSb_3_. (E) The band structures of Fe_4_Nb_4_NiSb_3_.

The band structure represents the collective electronic states in solid materials, formed through the overlap of atomic orbitals. The band structure critically influences the material’s electrical and optical characteristics. Figure 2B plots the band structure of intrinsic FeNbSb. Intrinsic FeNbSb exhibits an indirect band gap of 0.964 eV, with the conduction band minimum (CBM) and valence band maximum (VBM) residing at the Γ and R high-symmetry points, respectively. Figure 4 plots the band structure of derivative FeNbSb. Co/Ni-alloyed FeNbSb derivatives exhibit metallic characteristics due to the presence of energy bands crossing the Fermi level, contrasting with the semiconducting behavior of intrinsic FeNbSb. This is attributed to alloying, which induces modifications in lattice parameters and bond lengths of semiconductors. Furthermore, comprehensive density of states (DOS) and projected DOS (PDOS) plots for these systems are presented to probe the influence of alloying on *K*_IC_. The detailed discussion process can be found in section fracture toughness.

### Mechanical properties

As physical parameters characterizing the elastic properties of materials, elastic constants^28^^,^^29^ serve to quantify the deformation behavior of materials under external forces and provide crucial criteria for evaluating the mechanical stability of crystals.^30^ Among these parameters, Young’s modulus (*E*), shear modulus (*G*), and *v* represent key metrics for assessing the mechanical performance of materials. The *E* quantifies a material’s stiffness, where a high *E* indicates high rigidity and low deformability under stress. The *G* measures resistance to shear deformation, with a high *G* correlating to high toughness and resistance to shape distortion. *v* describes the ratio of transverse to axial strain under load. Together with *E* and *G*, it forms the core parameter system for the comprehensive characterization of a material’s elastic mechanical properties. All those *E*, *G*, and *v* can be deduced from the elastic stiffness coefficients (*C*_ij_). Table 2 presents the calculated independent elastic *C*_ij_ of FeNbSb and its alloyed derivative.

**Table 2:** The calculated independent elastic stiffness coefficients (*C*_ij_) of FeNbSb and its alloyed derivative

Crystal structure types	*C*_11_	*C*_44_	*C*_12_
FeNbSb	318.39 309.15^31^ 306.18^32^	66 65.51^31^ 43.61^32^	89.27 93.67^31^ 92.34^32^
Fe_4_CoNb_3_Sb_4_	314.44	82.20	99.42
Fe_3_CoNb_4_Sb_4_	319.47	76.46	95
Fe_3_NiNb_4_Sb_4_	306.32	77.64	97.54
Fe_4_Nb_4_CoSb_3_	325.71	51.02	109.02
Fe_4_Nb_4_NiSb_3_	304.29	50	109.02
Fe_2_Ni_2_Nb_4_Sb_4_	212.59	69.54	85.57
FeNi_3_Nb_4_Sb_4_	167.64	60.68	100.60

In the field of materials science, understanding the mechanical stability of materials is crucial for designing and developing novel materials with tailored properties. The Born stability criteria^33^^,^^34^ provide a theoretical framework for predicting the mechanical stability of various crystal structures, thereby guiding process selection and structural control during material synthesis. For HH FeNbSb alloy and its derivatives, which crystallize in cubic systems, the following mechanical stability conditions for cubic crystals must be satisfied^34^:

(Equation 3) $$C_{11}>0,C_{44}>0,C_{11}+2C_{12}>0,C_{11}-C_{12}>0\text{.}$$

Substituting the data from Table 2 into the stability criteria reveals that the elastic stiffness constants satisfy the Born criteria both before and after doping, demonstrating that both the intrinsic HH FeNbSb and its derivatives exhibit mechanical stability. Following the Voigt-Reuss-Hill (VRH) approximation,^35^^,^^36^^,^^37^^,^^38^ the bulk *B* and *G*^38^ were calculated from the derived *C*_ij_,

(Equation 4) $$B_{V}=B_{R}=\frac{C_{11}+2C_{12}}{3}\text{,}$$

(Equation 5) $$G_{V}=\frac{C_{11}-C_{12}+3C_{44}}{5}\text{,}$$

(Equation 6) $$G_{R}=\frac{5C_{44}\left(C_{11}-C_{12}\right)}{4C_{44}+3\left(C_{11}-C_{12}\right)}\text{.}$$

The subscripts “V” and “R” in quantities like *B*_V_, *B*_R_, *G*_V_, and *G*_R_ correspond to the Voigt and Reuss approximations, respectively—two core components of the VRH approximation method. The Hill model is established by taking the arithmetic mean of the values obtained from the Voigt model and the Reuss model.^26^

(Equation 7) $$B_{H}=\frac{B_{V}+B_{R}}{2}\text{,}$$

(Equation 8) $$G_{H}=\frac{G_{V}+G_{R}}{2}\text{.}$$

The *E* and *ν* were derived from the *B* and *G* through established theoretical formulations^39^:

(Equation 9) $$E=\frac{9GB}{G+3B}\text{,}$$

(Equation 10) $$v=\frac{3B-2G}{6B+2G}\text{.}$$

The Vickers hardness^40^^,^^41^ serves as a critical parameter for characterizing material hardness. The computational formula for Vickers hardness is expressed as follows^40^:

(Equation 11) $$H_{v}=0.92\;*k^{1.137}*G^{0.708}\text{,}$$

where *k* = *G*/*B*.

Cauchy pressure (*C*_P_) is a parameter in materials science and solid mechanics that primarily characterizes a material’s propensity for ductility or brittleness, particularly in predicting the mechanical behavior of intermetallic compounds or complex alloys. The Cauchy pressure can be derived from the elastic constants using the formula *C*_*P*_ = *C*_12_−*C*_44_.^42^ By substituting the values of *C*_12_ and *C*_44_ of the HH alloy FeNbSb and its derivative from Table 3 into the formula, the obtained Cauchy pressure *C*_P_ is positive. This suggests enhanced material ductility, favoring plastic deformation (dislocation slip) over brittle fracture.

**Table 3:** The calculated values of the bulk modulus *B*, shear modulus *G*, Young’s modulus *E*, Poisson’s ratio *v*, Cauchy pressure *C*_p_, *B*/*G*, Vickers hardness *H*_v_, and melting point *T*_m_ for FeNbSb and their derivatives

Crystal structure types	*B* (GPa)	*G* (GPa)	*E* (GPa)	*v*	*C*_p_ (GPa)	*B/G*	*Hv* (Pa)	*T*_m_ (°C)
FeNbSb	165.63 163^43^ 165.5^31^ 163.62^32^	89.83 79^43^ 80.3^31^ 63.04^32^	228.23	0.270	4.94	1.84	11.08	1,990.5 >1.200^44^
Fe_4_CoNb_3_Sb_4_	176.33	91.98	235.07	0.278	17	1.92	10.78	1,993.1
Fe_3_CoNb_4_Sb_4_	173.3	90.4	231.03	0.278	18	1.92	10.65	2,007.8
Fe_3_NiNb_4_Sb_4_	165.84	87.61	223.48	0.278	20	1.89	10.57	1,926.8
Fe_4_Nb_4_CoSb_3_	187.76	67.74	181.4	0.336	68	2.77	5.71	2,017.1
Fe_4_Nb_4_NiSb_3_	177.59	65.58	175.18	0.339	59	2.71	5.73	1,938.9
Fe_2_Ni_2_Nb_4_Sb_4_	130.94	62.77	162.37	0.293	16	2.09	7.47	1,944.0
FeNi_3_Nb_4_Sb_4_	117.27	47.74	126.11	0.321	40	2.46	5.11	1,650.9

The elastic moduli of these Fe-Nb-Sb-based compounds exhibit strong compositional dependence. Co incorporation enhances strength, resulting in higher *B*, *G*, and *E*. In contrast, Ni addition softens the lattice, reducing these moduli but promoting ductility. A notable exception is the Fe_4_Nb_4_MSb_3_ (M = Co, Ni) structure, which exhibits a unique combination of ultra-high *B* with a relatively lower *G*, leading to a high Pugh ratio (*B*/*G*) that predicts superior ductility. This highlights the crucial role of specific structural types in tailoring mechanical properties.

According to the Pugh criterion,^41^ materials are classified as ductile when their *B*/*G* ratio exceeds 1.75, whereas a ratio below 1.75 indicates brittle behavior. The calculated *B*/*G* ratios for FeNbSb, Fe_4_CoNb_3_Sb_4_, Fe_3_CoNb_4_Sb_4_, Fe_3_NiNb_4_Sb_4_, Fe_4_Nb_4_CoSb_3_, Fe_4_Nb_4_NiSb_3_, Fe_2_Ni_2_Nb_4_Sb_4_, and FeNi_3_Nb_4_Sb_4_ are 1.84, 1.92, 1.92, 1.89, 2.71, 2.77, 2.09, and 2.46, respectively. These results demonstrate that FeNbSb and its derivatives exhibit considerable ductility. By comparison, alloying significantly enhances material ductility, with Co/Ni substitutions at Sb sites demonstrating the most pronounced improvement in plastic deformability. These observations are further corroborated by the *ν* values^45^ of 0.270 (FeNbSb), 0.278 (Fe_4_CoNb_3_Sb_4_), 0.278 (Fe_3_NiNb_4_Sb_4_), 0.278(Fe_3_NiNb_4_Sb_4_), 0.339 (Fe_4_Nb_4_CoSb_3_), 0.336 (Fe_4_Nb_4_NiSb_3_), 0.293 (Fe_2_Ni_2_Nb_4_Sb_4_), and 0.321 (FeNi_3_Nb_4_Sb_4_). The calculated *ν* are all greater than 0.26, suggesting the material possesses excellent toughness.^46^ This correlation arises because *ν* is intrinsically linked to a material’s plasticity, where lower values typically indicate reduced capacity for plastic deformation. Furthermore, *ν* serves as an effective indicator for characterizing chemical bonding characteristics in solids.^47^ Distinct bonding types (e.g., metallic, covalent, ionic) induce fundamentally different transverse-to-longitudinal strain responses under mechanical loading, which are quantitatively reflected in their respective *ν* values. For the FeNbSb alloy system, the measured Vickers hardness values are 11∼12 GPa (FeNbSb), 10∼11 (Fe_4_CoNb_3_Sb_4_), 10∼11 (Fe_3_NiNb_4_Sb_4_), 10∼11 (Fe_3_NiNb_4_Sb_4_), 5∼6 (Fe_4_Nb_4_CoSb_3_), 5∼6 (Fe_4_Nb_4_NiSb_3_), 7∼8 (Fe_2_Ni_2_Nb_4_Sb_4_), and 5∼6 (FeNi_3_Nb_4_Sb_4_). While Co/Ni substitutions at Fe or Nb sites cause marginal reductions in Vickers hardness, those at Sb sites induce substantial decreases. Furthermore, the anisotropy indices calculated via Materials Studio provide quantitative insights into the materials' anisotropic characteristics, offering valuable guidance for material design and engineering applications.^48^^,^^49^

The melting point serves as a key metric for assessing material suitability in high-temperature environments. Fundamentally determined by the strength of interatomic bonding forces, it directly reflects high-temperature stability. This parameter can be derived from elastic constants via the following empirical relation:

(Equation 12) $$T_{m}=554+4.5\left(\frac{2C_{11}+C_{33}}{3}\right)\text{.}$$

As listed in Table 3, calculations of the melting point indicate that intrinsic FeNbSb is a high-temperature alloy material with a melting point up to 1,900 K. Relevant studies also confirm its role as a high-temperature thermoelectric material. The effects of Ni and Co alloying on the melting point are contrasting: Co alloying enhances the melting point of FeNbSb, particularly when Co substitutes Sb, reaching 2,017 K. In contrast, Ni alloying reduces the melting point, which decreases further with increasing Ni content. This phenomenon likely arises from lattice distortions due to atomic radius variation, which alters the local bonding environment.

### Fracture toughness

Griffith^50^ established an energy-based theoretical framework to explain material fracture occurring below theoretical strength limits. This pioneering theory analyzes stress concentration at crack tips and defines the energy threshold required for crack propagation, ultimately formulating the critical condition for fracture initiation known as the Griffith Crack Propagation Criterion. The Griffith Crack Propagation Criterion stands as the fundamental achievement of Griffith’s fracture theory.^51^^,^^52^ This theoretical framework establishes the instability criterion for crack propagation through energy balance principles: crack extension and material fracture occur when *G*_Griffith_ ≥ *G*_IC_, whereas crack stability is maintained when *G*_Griffith_ < *G*_IC_. Here, *G*_Griffith_ characterizes the energy released per unit area of crack advancement and serves as a critical metric for describing crack propagation behavior. *G*_IC_ represents the material’s critical energy release rate, intrinsically related to its surface energy and other fundamental properties. The formula of *G*_IC_ is expressed as follows^53^:

(Equation 13) $$G_{IC}=\frac{E_{crack}-E_{initial}}{A_{crack}}\text{.}$$

Here, *E*_crack_ represents the crack-associated energy, which accounts for the energy variation during crack formation or propagation. *E*_initial_ denotes the energy of the initial crystal structure, also measured in electron volts. *A*_crack_ corresponds to the crack’s cross-sectional area in square angstroms.

*K*_IC_, as a crucial mechanical property parameter characterizing a material’s resistance to crack propagation and brittle fracture, is fundamentally correlated with Griffith’s fracture theory. This parameter quantitatively reflects a material’s capacity to withstand external loading without catastrophic failure in the presence of flaws or cracks, typically represented by the critical stress intensity factor with units of Mpa・m^1/2^. *K*_IC_ demonstrates a direct positive relationship with crack resistance; materials exhibiting higher *K*_IC_ values possess superior fracture resistance. For alloy materials subjected to Mode I loading (where the load is perpendicular to the cleavage plane), the *K*_IC_ can be specifically defined as the critical stress intensity factor.

For cubic crystal systems like FeNbSb and its derivative alloys, the calculation incorporates specific crystallographic orientation considerations. We selected the [001] direction for cleavage plane orientation and introduced the crack accordingly. To compute the *K*_IC_ of a crack, obtaining the elastic constants—such as *E* and *ν* —of the two-dimensional (2D) fracture plane is essential. When reducing the dimensionality from 3D to 2D, the constraints or boundary conditions along the thickness direction are altered, leading to corresponding changes in the strain energy. By analyzing these strain energy variations and incorporating 2D geometric characteristics (such as thickness *h*), we can derive the relationship between 2D and 3D elastic constants as follows:

(Equation 14) $$C_{ij}\left(2D\right)=C_{ij}\left(3D\right)*h$$

By calculating the elastic constants of 2D materials, we derive the *E* and *ν* required for *K*_IC_ computation. These parameters are then incorporated into the following theoretical formula^54^^,^^55^:

(Equation 15) $$K_{\text{IC}}=\sqrt{\frac{G_{IC}*E}{1-v^{2}}}\text{,}$$

where *G*_IC_ represents the fracture energy of the unit cell model (in J/m^2^), *E* denotes the elastic modulus of the unit cell model (in GPa), and *ν* is Poisson’s ratio (dimensionless).

An alternative approach for estimating *K*_IC_ employs the following relation^56^^,^^57^:

(Equation 16) $$K_{\text{IC}}=V_{0}^{1/6}G{\left(B/G\right)}^{1/2}\text{,}$$

where *V*_0_ denotes the atomic volume and *B* and *G* represent the bulk and shear moduli, respectively.

To evaluate damage tolerance, the brittle index M is calculated as,^57^^,^^58^

(Equation 17) $$M=H_{V}/K_{\text{IC}}\text{,}$$

where *H*_V_ corresponds to Vickers hardness. A lower M value indicates superior damage tolerance. The key parameters required for calculating *K*_IC_ using two methods, along with the resulting values, are summarized in Table 4. The close agreement between the *K*_IC_ values obtained from the two methods confirms the reliability of the computational approaches.

**Table 4:** The calculated crack area *A*_crack_, 2D shear modulus *G*^2D^, Poisson’s ratio *v*, Young’s modulus *E*^2D^, critical energy release rate *G*_IC_, *K*_IC_, and brittle index M of FeNbSb and their derivatives

Compounds	*A*_crack_ (Å^2^)	*G*^2D^ (GPa)	*v*	*E*^2D^ (GPa)	*G*_IC_ (J/m^2^)	*K*_IC_ (Mpa·m^1/2^)		M	
FeNbSb	5.93293	114.559	0.21898	279.291	1.71145	2.836^a^	1.964^b^	3.91^a^	5.65^b^
Fe_4_CoNb_3_Sb_4_	5.80748	107.504	0.24024	266.664	1.59479	2.689^a^	2.043^b^	4.01^a^	5.56^b^
Fe_3_CoNb_4_Sb_4_	5.84682	112.231	0.22921	275.915	1.51469	2.658^a^	2.020^b^	4.01^a^	5.43^b^
Fe_3_NiNb_4_Sb_4_	5.91271	104.386	0.24153	259.197	1.82544	2.837^a^	1.944^b^	3.73^a^	5.23^b^
Fe_4_Nb_4_CoSb_3_	5.74071	102.993	0.26877	261.351	1.41129	2.524^a^	1.810^b^	2.26^a^	3.15^b^
Fe_4_Nb_4_NiSb_3_	5.7388	97.635	0.26376	246.776	1.46979	2.499^a^	1.730^b^	2.29^a^	3.31^b^
Fe_2_Ni_2_Nb_4_Sb_4_	5.968	63.51	0.28699	163.473	1.70856	2.208^a^	1.471^b^	3.38^a^	5.08^b^
FeNi_3_Nb_4_Sb_4_	4.98	33.521	0.37503	92.1861	1.77008	1.744^a^	1.220^b^	2.93^a^	4.19^b^

a The first calculation method obtained from the *G*_IC_, *E*, and *v*.

b The second calculation method obtained from the *B* and *G*.

**Figure 5:**
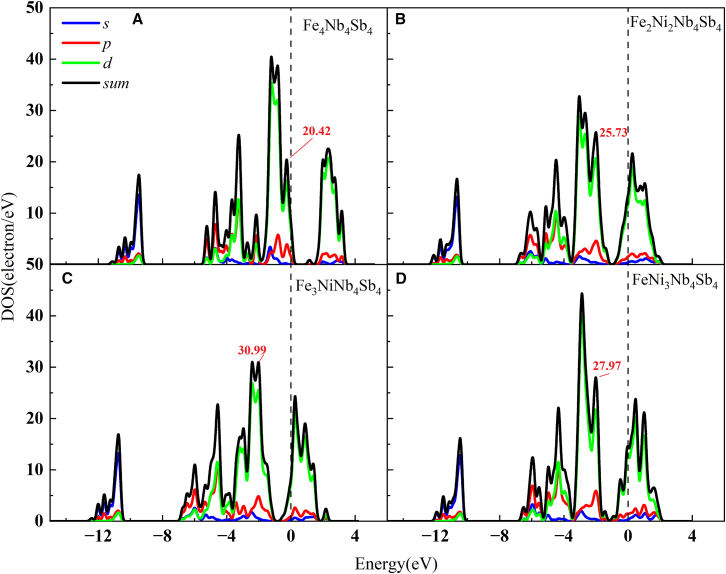
DOS and PDOS of FeNbSb and its derivatives alloyed with Ni atoms (A) The DOS and PDOS of FeNbSb. (B) The DOS and PDOS of Fe_3_NiNb_4_Sb_4_. (C) The DOS and PDOS of Fe_2_Ni_2_Nb_4_Sb_4_. (D) The DOS and PDOS of FeNi_3_Nb_4_Sb_4_.

*K*_IC_ improvement was observed exclusively in cases where Co substituted for Fe and Nb, i.e., the undoped alloy exhibits a *K*_IC_ of 1.964 Mpa·m^1/2^, which increases to 2.02 Mpa·m^1/2^ and 2.043 Mpa·m^1/2^ when Co replaces Fe and Nb atoms. However, both the cases where Co replaces Sb and Ni replaces Fe, Nb, and Sb result in a decrease in *K*_IC_. This trend is primarily governed by the atomic radius strategy. When the radius of the doping atom (*R*_doping_) falls between those of the host atoms (*R*_X_<*R*_doping_<*R*_Y_), the alloying effect enhances toughness. Conversely, the use of dopants with smaller atomic radii (*R*_doping_<*R*_X_<*R*_Y_) leads to diminished *K*_IC_,^59^^,^^60^ which is consistent with our observations. Furthermore, Ni substitution for Fe gradually reduced *K*_IC_ with increasing Ni concentration. This decline is attributed to the enhanced bonding peak on the left side of the Fermi level as the Ni percentage rises (see Figure 5). The corresponding bonding peaks are 20.42, 30.99, 25.73, and 27.97, respectively. This phenomenon aligns with the behavior reported in Huang et al.^61^ Our results reaffirm that alloying with larger-radius elements can effectively enhance toughness, offering a viable strategy for material toughening.

## Discussion

In this study, we systematically investigated the structural stability, electronic properties, mechanical behavior, and *K*_IC_ of intrinsic HH FeNbSb and its Co/Ni-doped derivatives using first-principles calculations. The key findings are summarized as follows.

All doped systems except Fe_4_NiNb_3_Sb_4_ exhibit dynamic and thermodynamic stability, with Co/Ni substitutions at Fe sites being the most energetically favorable due to similar atomic radii and electronic configurations. Electronic structure calculations reveal that doping eliminates the semiconducting band gap of intrinsic FeNbSb (0.964 eV), resulting in metallic character with bands crossing the Fermi level.

Mechanical property analysis demonstrates that all stable structures satisfy the Born criteria for cubic crystals. Doping enhances resistance to volumetric deformation, as evidenced by increased bulk moduli. The Pugh criterion (*B/G* > 1.75) and *ν* > 0.26 confirm significant ductility across all systems, with Co/Ni substitutions at Sb sites exhibiting the highest ductility (*B/G* ≈ 2.7–2.8).

Critically, the calculations reveal that Co substitution at Fe and Nb sites improves *K*_IC_ up to 2.043 MPa m^1/2^ compared to 1.964 MPa m^1/2^ for intrinsic FeNbSb, while Ni doping and Co substitution at Sb sites reduce crack propagation resistance. This toughness enhancement is governed by an atomic radius strategy: dopants with radii intermediate between host atoms (*R*_X_ < *R*_dopant_ < *R*_Y_) promote toughness, whereas smaller-radius dopants diminish it. The observed trend correlates with bonding characteristics near the Fermi level, where increased bonding peak intensity corresponds to reduced toughness.

These results provide fundamental insights into toughening mechanisms of HH alloys and demonstrate that targeted Co doping at Fe/Nb sites offers a viable strategy for enhancing fracture resistance, while maintaining excellent thermodynamic stability and ductility. This work establishes atomic-radius-based design principles for developing damage-tolerant thermoelectric materials capable of withstanding mechanical loads in high-temperature applications.

### Limitations of the study

While this study provides fundamental insights into the fracture toughness and doping effects in FeNbSb-based HH alloys through first-principles calculations, several limitations should be acknowledged. First, our computational models are based on idealized, defect-free single crystals at 0 K. Real-world materials contain grain boundaries, dislocations, and other defects, which can significantly influence crack initiation and propagation, potentially altering the fracture toughness in polycrystalline forms. Second, the calculations do not account for temperature effects on the elastic constants and fracture behavior. Given the intended high-temperature applications of these materials, the potential for thermal softening or changes in ductile-to-brittle transition temperatures with doping remains an important area for future experimental investigation. Finally, the predictions made herein, particularly the enhanced toughness with Co doping at Fe/Nb sites, await experimental validation through the synthesis of these doped phases and subsequent mechanical testing.

## Resource availability

### Lead contact

Further requests for computational data, detailed methodologies, or collaborative inquiries should be directed to the lead contact, Shao-Bo Chen (shaobochen@yeah.net).

### Materials availability

This study did not generate new physical materials.

### Data and code availability

• All other data reported in this paper will be shared by the lead contact upon request.
• This paper does not report original code.
• Any additional information required to reanalyze the data reported in this paper are available from the lead contact upon request.

## Acknowledgments

This work was supported by the Training Program of Innovation and Entrepreneurship for College Students (grant no. S202310667157), the Scientific Research Training Program for College Students (grant no. asxysrt202327), the 10.13039/501100001809National Natural Science Foundation of China (grant no. 12364017), the Ph.D. Fund Project of 10.13039/501100020768Anshun University (grant no. asxybsjj202317), and Anshun City’s 2025 Basic Research Project of the Science and Technology Bureau (grant no. [2025]01).

## Author contributions

Conceptualization, S.-B.C. and J.-H.W.; methodology, S.-B.C., J.-H.W., and W.-J.L.; software, T.-H.G. and W.-J.Y.; formal analysis, S.-B.C. and J.-H.W.; data curation, S.-B.C.; writing – original draft, S.-B.C. and J.-H.W. writing – review and editing, S.-B.C. and W.-J.Y.; funding acquisition, S.-B.C.; supervision, S.-B.C., J.-H.W., and W.-J. Y.

## Declaration of interests

The authors declare no competing interests.

## STAR★Methods

### Key resources table

**Table:** 

REAGENT or RESOURCE	SOURCE	IDENTIFIER
**Software and algorithms**		

Materials Studios	M D Segall et al.^62^	https://www.castep.org/

### Method details

In this work, all the calculations were simulated using the CASTEP code^62^ based on the density functional theory (DFT). For the exchange-correlation function in Materials Studio, the Generalized Gradient Approximation (GGA)^63^ with the Perdew-Burke-Ernzerhof (PBE) method was employed to evaluate the electronic properties and mechanical performance of the materials. The cutoff energy for the plane-wave basis set was set to 400 eV with a 12×12×12 k-point mesh for Brillouin zone sampling.^64^ The convergence criteria are adjusted using the Broyden-Fletcher-Goldfarb-Shanno (BFGS) scheme.^65^ The interactions of valence electrons with ionic cores were modeled using ultra-soft pseudo-potentials (USPP). The convergence criteria for electronic and ionic relaxations were set at 10^-6^ eV and 0.001 eV/Å, respectively. To verify the thermodynamic stability of the FeNbSb and its derivative alloyed with Co and Ni, phonon dispersion curves were computed via the CASTEP code. The calculation processes and formulas for other properties (formation enthalpy, site occupation energy, Vickers hardness, melting point, *K*_IC_, etc.) are detailed in the following text.

### Quantification and statistical analysis

Our study does not include statistical analysis or quantification.
